# Choroidal Thickness and Primary Open-Angle Glaucoma—A Narrative Review

**DOI:** 10.3390/jcm11051209

**Published:** 2022-02-23

**Authors:** Alice Verticchio Vercellin, Alon Harris, Ari M. Stoner, Francesco Oddone, Kristen Ann Mendoza, Brent Siesky

**Affiliations:** 1 Department of Ophthalmology, Icahn School of Medicine at Mount Sinai, New York, NY 10029, USA; alice.verticchio@mssm.edu (A.V.V.); alon.harris@mssm.edu (A.H.); 2 Department of Ophthalmology, Indiana University School of Medicine, Indianapolis, IN 46202, USA; armstone@iu.edu; 3 IRCCS-Fondazione Bietti, 00198 Rome, Italy; oddonef@gmail.com; 4 New York Eye & Ear Infirmary of Mount Sinai, New York, NY 10003, USA; kristenann.mendoza@mountsinai.org

**Keywords:** choroid, choroidal thickness, open-angle glaucoma, imaging, optical coherence tomography

## Abstract

The choroid provides the majority of blood flow to the ocular tissues and structures that facilitate the processes of retinal metabolism responsible for vision. Specifically, the choriocapillaris provides a structural network of small blood vessels that supplies the retinal ganglion cells and deep ocular tissues. Similar to retinal nerve fiber layer thickness, choroidal thickness (CT) has been suggested to represent a quantifiable health biomarker for choroidal tissues. Glaucoma is a disease with vascular contributions in its onset and progression. Despite its importance in maintaining ocular structure and vascular functionality, clinical assessments of choroidal tissues have been historically challenged by the inaccessibility of CT biomarker targets. The development of optical coherence tomography angiography and enhanced depth imaging created a framework for assessing CT and investigating its relationship to glaucomatous optic neuropathy onset and progression. Pilot studies on CT in glaucoma are conflicting—with those both in support of, and against, its clinical utility. Complicating the data are highly customized analysis methods, small sample sizes, heterogeneous patient groups, and a lack of properly designed controlled studies with CT as a primary outcome. Herein, we review the available data on CT and critically discuss its potential relevance and limitations in glaucoma disease management.

## 1. Introduction

Primary open-angle glaucoma (OAG) represents the second leading cause of blindness worldwide and is estimated to impact 112 million persons by 2040 [1]. OAG is a primary chronic optic neuropathy characterized by an open angle of the eye, loss of retinal ganglion cells, and associated progressive and irreversible vision loss. OAG is responsible for close to three-quarters of all glaucoma cases worldwide and is the most common form of glaucoma in populations of both European (ED) and African descent (AD) [2,3]. Importantly, OAG disproportionately impacts people of AD, with AD populations having up to six times the prevalence of OAG compared to ED, an earlier onset of the disease, a more severe course, and a faster progression [4,5].

Currently, reduction of intraocular pressure (IOP) remains the only approved therapeutic route for OAG, as large population-based studies have shown that elevated IOP is associated with glaucoma prevalence, incidence, and progression [4]. The main goals of OAG treatment are to lower IOP into a target range to prevent progressive visual field and optic nerve and retinal nerve fiber layer (RNFL) damage and preserve visual function and quality of life [3]. There are a variety of methods to lower IOP, including pharmacologic, laser, and surgical management. The four major classes of IOP-lowering medications are represented by beta-blockers, adrenergic agonists, carbonic anhydrase inhibitors, and prostaglandin analogs [6]. Generally, prostaglandin analogs are the first-line pharmacologic therapy, given their lower side effect profiles, while the other classes of topical medications are generally used as second-line agents behind or in conjunction with prostaglandin analogs if monotherapy is not successful in sufficiently lowering IOP [7]. Alternatively, selective laser trabeculoplasty, a type of treatment that increases aqueous humor outflow through the trabecular meshwork using argon or solid-state lasers, may be considered a first-line therapy for patients who have severe disease or difficulty adhering to medication regimens [7]. Among surgical options, the most common procedure is represented by trabeculectomy, which creates an alternate filtration route for aqueous humor to drain from the eye to under the conjunctiva. Alternatives to trabeculectomy include implantation of mechanical shunts and newer minimally invasive glaucoma surgeries (MIGS), characterized by a lower level of invasiveness compared to the traditional filtering surgery techniques, and reduced ocular surface toxicity compared to the long-term therapy with IOP-lowering medications [8].

A reduction of IOP, however, has not cured glaucoma. A high percentage of individuals with elevated IOP do not develop glaucoma, and many persons with OAG have relatively low IOP. Additionally, reduction of IOP does not always arrest the disease process, thus, highlighting the fact that OAG represents a multifactorial pathology with other risk factors involved in its onset and progression [4]. Importantly, a multitude of research has identified impaired ocular blood flow in OAG patients, with vascular deficits reported in retinal, choroidal, and optic nerve head tissues [4]. The vast majority of blood flow within the eye occurs in the choroidal tissues, including the choriocapillaris, a structural network of small vessels that supplies the retinal ganglion cells with perfusion, thereby facilitating cellular metabolism. Historically, the critical choroidal tissues have been difficult to directly image and quantify; however, recent advances in ocular imaging have allowed some of the first biomarkers of choroidal tissues to be recorded in living human subjects.

As with RNFL thickness, the choroid tissue depth is largely seen as a biomarker of healthy functional tissue margins. Physiologically, the choroid encompasses the majority of blood flow to the eye, and the retinal pigment epithelium distinctly depends on the choriocapillaris for its perfusion and metabolism [9]. Despite its obvious importance in maintaining ocular structure and vascular functionality, choroidal thickness (CT) and its relationship to glaucomatous optic neuropathy onset and progression are currently without consensus. The relative weight of vascular and/or structural insult of choroidal tissues to the glaucomatous process has been discussed for many decades; however, it has been historically difficult to image and quantify choroidal tissue due to its deep orbital anatomical location. Only over the past decade have advances in optical coherence tomography (OCT), OCT angiography (OCTA), and applications of enhanced depth imaging (EDI) allowed for quantification of CT in human subjects. EDI-OCTA has provided a methodology for pilot studies to quantify CT and investigate its association with certain aspects of glaucoma. However, the custom and highly varied applications of quantifying EDI CT biomarkers, small study sample sizes, a lack of diversity in patient populations, and a void of comprehensive assessments in relation to CT biomarkers to date have failed to provide consensus. In chronic progressive ocular diseases, such as OAG, a better understanding of CT and its importance in ocular physiology and pathology is strongly warranted.

The currently available literature on CT and glaucoma is diverse and conflicting, providing evidence both for and against CT as an independent risk factor for glaucoma. Herein, we present and review the available literature on CT and OAG, with an emphasis on available methods to assess CT, specific factors influencing its measurement, and discussion on choroidal thinning as reported in glaucomatous eyes with suggestions for needed follow-up investigations.

## 2. Materials and Methods

PubMed, Embase, Ovid, Scopus, and Trip searches were conducted through 31 December 2021 to evaluate all pertinent articles, abstracts, and ongoing research projects. The searched keywords included glaucoma, primary open-angle glaucoma, open-angle glaucoma, choroid, choroidal thickness, optical coherence tomography, optical coherence tomography angiography, enhanced depth imaging, perfusion, blood flow, retina, and choroidal imaging. The same keywords were used in all search software, articles were screened for relevance, and reference lists of relevant articles were also searched and cross-referenced for other relevant articles. Data were collected and organized using Microsoft Word (version 16.30), Microsoft Excel (version 16.30), and EndNote (X8.2).

## 3. Discussion

### 3.1. Methods of Assessment of Choroidal Thickness

As the importance of CT measurements has been highlighted in recent years, multiple methodologies have been created in order to assess it, each with specific advantages and modality limitations. The first measurement of CT was performed over 100 years ago on casts of autopsy eyes; however, this method itself had unknown effects on the choroid and CT measurements [10]. Modern methods of assessment include indocyanine green angiography (ICG) [11,12], B-scan ultrasonography [13], and more recently, applications of EDI-OCT [14] and swept-source OCT (SS-OCT) [15].

Historically, when attempting to image the choroid, B-scan and ICG provided a qualitative observation of vascular structures, but these methods lack objective cross-sectional information [14]. Newer applications of OCT have more recently allowed for high-resolution, direct, and non-invasive imaging of the retina, pilot assessment, and quantification of CT in human subjects [14]. By employing image averaging and setting a zero-line adjacent to the choroid, EDI-OCT has been able to provide a somewhat detailed, measurable image of the choroid [14]. The choroidal-scleral boundary can be difficult to discern by EDI-OCT and typically relies on manual measurements and localization points. SS-OCT has therefore been used in recent years to measure the choroidal thickness and overcome these challenges [15,16]. Rather than relying on silicone-based, line scan, charge-coupled device cameras, such as in spectral-domain OCT (SD-OCT), SS-OCT devices use a tunable laser and photodetectors, reducing light scatter by the retinal pigment epithelium and enabling better visualization of deeper ocular structures, including the choroid [17]. SS-OCT is also capable of providing a wide field scan (12 × 9 mm). This approach allows for imaging of the macula and optic nerve head simultaneously, having the advantage of acquiring measurements of peripapillary and macular CT in the same scan.

An interesting study from Komma et al. [18] investigated macular and peripapillary CT in 17 OAG patients and 20 normal subjects using both SD-OCT and SS-OCT. The authors found that CT measurements assessed by SD-OCT were lower than those assessed by SS-OCT, possibly caused by a better delineation of the junction between the sclera and choroid with SS-OCT. Therefore, CT measurements taken with different imaging devices may not be comparable, and when analyzing the results from different studies assessing CT, it is crucial to consider the specific methodology used for its assessment.

### 3.2. Choroidal Thickness Differences between Healthy and Glaucomatous Eyes

The available literature on CT and patients with glaucoma is conflicting, providing evidence both for and against CT as a risk factor [19,20,21,22,23,24,25,26,27,28,29,30,31,32,33,34,35]. In general, the literature contains a diverse set of measurement approaches prior to EDI, and subsequently, there is a lack of properly designed and powered trials to appropriately assess CT over the course of glaucomatous disease. Historically, glaucoma was thought to be associated with a thinner choroid, as demonstrated by multiple studies [19,20,21,22,23], yet several contrasting studies have shown that healthy and glaucomatous eyes have similar CT [24,25,26,27,28,29]. A summary of relevant studies investigating CT in OAG patients and healthy subjects, and their findings, are shown in Table 1.

The anatomical location of the CT measurement—macular of peripapillary—represents an important factor to consider when evaluating its role in OAG. The results from a meta-analysis by Wang and Zhang in 2014, across sixteen studies on 875 OAG patients and 871 healthy controls, found no significant differences in CT between glaucomatous patients and healthy subjects, with a pooled weighted mean difference (WMD) of −7.36 µm (95% confidence interval [CI]: −24.39 to 9.67, *p* = 0.397) for subfoveal CT, and of 6.67 µm (95% CI: −2.45 to 15.79, *p* = 0.152) for mean macular CT [31]. The authors also evaluated data for the average peripapillary CT in six studies and found a significant difference with a pooled estimate of −8.15 µm (95% CI: −16.16 to −0.13, *p* = 0.046). However, the authors highlighted in their article how an instrument error could have caused this difference since the OCT precision was limited to 10 µm. Considering these results, the authors concluded that their study did not find a significant association between CT and OAG. These results were confirmed by a more recent meta-analysis published in 2016 by Zhang et al., utilizing twenty-two case-control or cross-sectional studies [36]. The authors found no significant difference in CT between OAG patients and controls both for subfoveal CT (WMD = −7.94; 95% CI: −26.01 to 10.13, *p* = 0.389) and for peripapillary CT (WMD = −14.24; 95% CI: −30.20 to 1.73, *p* = 0.08); thus, concluding that CT may not represent an appropriate parameter to assess glaucomatous damage [36].

In summary, there is evidence both in support and against the possible use of CT as a diagnostic biomarker, and its association with the OAG disease process requires clarification. A significant limitation of the current literature is a lack of properly designed and statically powered clinical trials with CT as a primary endpoint matched with appropriate controls. Most currently available studies are secondary analyses not designed with CT as a primary control outcome, and the literature, in general, is anemic in relation to CT and OAG. While some pilot studies provide evidence for CT as a diagnostic biomarker, the heterogeneous nature of the studies, conflicting results, and differing methods of CT assessment prevent conclusion. Further, anatomical norms and differences in baseline CT are not well established; thus, careful consideration of race, gender, and age are critical factors to consider when creating a CT protocol. Accounting for IOP, length of OAG, corneal thickness, and other variables may also need to be considered when designing longitudinal studies on CT. Importantly, a better understanding of CT and its relationship to retinal ganglion cell survival and preservation of visual function over the course of glaucoma progression is currently missing and required to actualize CT in glaucoma management.

### 3.3. Factors Influencing Choroidal Thickness

Several physiological factors intertwined with CT play a role in OAG pathophysiology, such as age, IOP, ocular perfusion pressure, axial length, and corneal thickness [23,28,37]. As age, lower perfusion pressures, and differences in ocular structure may alter OAG risk, CT and its relationship with each factor become important to understand. An important question to consider is, “Does a thinner CT biomarker indicate worse visual functionality in OAG, or does a thinning choroid represent tissue loss and anemic demand?”

Current literature on CT and visual function is mixed and lacks properly designed studies with primary visual outcomes and matched controls. In a study by Maul et al., which did not find a difference in CT between glaucomatous eyes and non-glaucomatous eyes, there was additionally no correlation with structural disease severity as measured by RNFL thickness and/or visual field mean deviation [28]. Similar results were obtained by a recent study from Karaca et al., who did not find any significant difference in macular and peripapillary CT between healthy and glaucomatous eyes with different severity of functional damage [38]. Further studies are therefore needed to investigate the association between structural characteristics of the choroid and functional vision loss in glaucoma.

Results from sub-analyses of the Beijing Eye Study 2011, a large population-based cross-sectional study based in northern China on 3468 participants, shed further light on the correlations between CT assessed by SD-OCT and other ocular and systemic parameters [29,37]. In detail, in this adult Chinese population, the authors found that thicker subfoveal choroid was significantly associated in univariate analysis with several ocular parameters (better best corrected visual acuity, BCVA; shorter axial length; hyperopic refractive error; shorter anterior chamber depth; thinner lens; steeper cornea; higher IOP). A thicker choroid was also significantly correlated with patient demographics and systemic parameters (younger age, male gender, greater body height, weight, body mass index, rural region of habitation, higher diastolic and mean blood pressure, presence of arterial hypertension, higher ocular perfusion pressure, higher serum concentrations of cholesterol and glucose, smoking, higher number of package years of cigarettes, higher alcohol consumption, less aspirin intake, and higher frequency of reported snoring). Interestingly, the strong association between a thicker subfoveal choroid and BCVA was also shown after adjusting for other factors, such as younger age, male gender, longer axial length, and higher corneal curvature radius (all *p* < 0.001) [37]. In another analysis of the same cohort, the authors evaluated the peripapillary CT and its associations and found that thicker peripapillary CT was associated with younger age, shorter axial length, better BCVA, and a higher prevalence of early and intermediate age-related macular degeneration [29]. Instead, peripapillary CT was not significantly associated with blood pressure, blood lipid concentration, IOP, and presence of major ocular diseases, such as glaucoma, diabetic retinopathy, or retinal vein occlusions [29]. Thus, it is important to highlight how the association between CT and other parameters, including systemic and ocular diseases—such as glaucoma—may be affected by the anatomical location of the CT measurement, including analysis placement at the macular versus peripapillary CT.

### 3.4. Potential Mechanisms behind the Relationship between CT and Glaucoma

There is currently a void in the understanding of OAG pathophysiology, the relative weight of risk factors, and the potential role of a CT in monitoring the disease process. Reduction of IOP remains the only currently approved therapeutic route for OAG, while vascular health has also been associated with OAG onset and progression. The choroid encompasses the vast majority of perfusion to ocular tissues, and its thickness, similar to RNFL, is likely an important biomarker of functional health. Several potential mechanisms have been suggested to explain the observed relationships between CT and glaucoma, even if not uniform in agreement. Lee et al., found that the mean juxtapapillary CT (JPCT) was significantly smaller in OAG patients compared to healthy subjects (*p* = 0.0410). The authors also found that in OAG patients, there was a significantly stronger topographical association between JPCT and the width of the inferotemporal β-zone parapapillary atrophy with an intact Bruch’s membrane, but not with the width of the inferotemporal β-zone parapapillary atrophy devoid of the Bruch’s membrane. The authors, therefore, suggested that in glaucoma, JP choroidal atrophy and the formation of inferotemporal β-zone parapapillary atrophy may share a common—possibly vascular—pathogenetic pathway [39]. In another study on OAG patients with β-zone peripapillary atrophy, parapapillary deep layer microvascular dropout was found to be associated with thinner total choroidal thickness, thus supporting the concept of the association between thinning of the choroid and choriocapillaris loss [15]. It has also been proposed that choroidal thinning itself may lead to reduced choroidal circulation, causing a subsequent hemodynamic insufficiency of the prelaminar region since the prelamina is supplied mainly by recurrent choroidal arterioles and short posterior ciliary arteries [40,41]. Notably, Duijm et al., found slower choroidal circulation in normal-tension glaucoma patients compared with control subjects [42].

Finally, increased IOP as a mechanical force has been considered as a potential mechanism behind reported associations of CT and glaucoma. Higher IOP may physically impact choroidal tissues themselves and/or reduce overall vascular perfusion within small vessels and tissue volume accordingly. In this regard, it is important to highlight that the relationship between CT and glaucoma may be influenced by the therapeutical interventions aimed to reduce the disease progression by lowering the IOP in glaucoma patients, medically or in surgery. Specifically, the lowering of IOP attributed to trabeculectomy has been associated with an increase in CT in several studies, and it has been suggested that the decrease in IOP with trabeculectomy may cause an increase in CT as the IOP force on the choroid is reduced [43,44,45]. CT has also been found to increase linearly with IOP lowering after undergoing either trabeculectomy or laser trabeculoplasty with a mean choroidal vessel thickness increase of 1.5 um for every 1 mmHg decrease in IOP (*p* < 0.0001) and choroidal interstitial thickness increase of 1.3 um for every 1 mmHg change in IOP (*p* < 0.0001) [46]. In addition, some glaucoma medications are known to alter the hemodynamics of ocular tissues and may, therefore, also impact CT [47]. Future prospective, large sample longitudinal studies are needed to understand how different medication categories may impact CT.

## 4. Conclusions and Future Directions

The management of glaucoma remains challenging due to its heterogeneous presentation and lack of non-IOP therapeutic targets. The choroid provides the vast majority of ocular perfusion to ocular structures, and lower hemodynamic biomarkers have been long identified in OAG patients. Until recently, the direct imaging of deep choroidal tissues has been difficult to impossible, with recent advances in EDI OCTA and other custom applications providing the first quantitative measures of CT in humans. While the ability to visualize deeper ocular tissues and structures holds great promise for understanding disease processes in the eye, improving diagnosis, and identifying novel treatment targets, current literature on CT in glaucoma is sparse, with conflicting results and uncertain relationships. Specifically, peripapillary and macular CT thinning has been reported in some patients with OAG; however, other pilot works failed to confirm these findings in glaucoma patients. The field is novel, and the applications of CT measurements have yet to be fully developed. The exact relationships of CT, its physiological meaning, and relationship to OAG risk remain undefined. The available literature contains only pilot and sub-analysis studies on CT, while large, properly designed, and statistically powered case-controlled trials on CT in OAG are currently missing.

Importantly, there are currently no normative baseline values established for CT, and its relationship with age, sex, race, IOP, and hemodynamics are uncertain. In addition, no comparative, controlled data are available on the multi-dimensional relationships of CT, IOP, structural and functional biomarkers of OAG, and ocular hemodynamics and metabolism in OAG. Confounding factors such as age, sex, race, and body mass index are important to consider when properly designing a study on CT based upon their influence on other imaging modalities. Specifically, there are no baseline or comparative data on potential differences in CT and its relationships to OAG in patients of AD and ED. With acknowledgment of current limitations in approach and comparative data, biomarkers of CT have enough justification for being examined more critically. When considering diagnostic applications, it will be important to investigate CT across population groups and between genders to account for unknown confounding physiological variables. It is also important to acknowledge that further studies are needed to investigate CT in different types of glaucoma with different mechanisms of pathophysiology. These include primary forms (such as primary angle-closure glaucoma) and secondary forms (such as pseudo-exfoliative and pigmentary glaucoma). It is likely that specific pathophysiologic mechanisms driving different glaucoma types have different relationships with CT. Going forward, careful consideration, open-access publication, and open sourcing of CT methodology techniques will be pivotal for its potential uptake and utilization in the management of glaucoma.

## Figures and Tables

**Table 1:** Summary of relevant studies investigating choroidal thickness measurement in open-angle patients and healthy subjects. CT: choroidal thickness; NTG: normal-tension glaucoma; OAG: primary open-angle glaucoma.

Author	Study Population	Measurement	Method	Mean CT (um)	*p*-Value
Li 2013 [24]	OAG patients with unilateral visual field loss (31) vs. healthy controls (31)	Global mean peripapillary CT	Enhanced depth imaging optical coherence tomography	OAG eyes with visual field loss: 154.3 ± 69.7 Eyes of healthy controls: 154.2 ± 60.9	0.994
Lee 2016 [23]	NTG patients (96) vs. healthy controls (48)	Average juxtapapillary CT	Swept-source optical coherence tomography	NTG patients: 107.66 ± 37.65 Healthy controls: 135.56 ± 42.66	<0.001
Wang 2014 [31]	OAG patients (52) vs. healthy controls (76)	Average macular CT	Enhanced depth imaging optical coherence tomography	OAG patients: 221.5 ± 44.1 Healthy controls: 224.1 ± 43.7	0.747
Mwanza 2011 [26]	OAG patients (56) vs. NTG patients (20) vs. healthy controls (38)	Subfoveal CT	Enhanced depth imaging optical coherence tomography	Glaucoma patients (OAG and NTG combined): 216.16 Healthy controls: 214.68	0.92
Cennamo 2012 [33]	OAG patients (16) vs. healthy controls (21)	Subfoveal CT	Spectral-domain optical coherence tomography	OAG patients: 411.56 ± 33.6 Healthy controls: 343.8 ± 29.06	<0.001
Ehrlich 2011 [27]	OAG patients (31) vs. glaucoma suspects (39)	Global peripapillary CT	Spectral-domain optical coherence tomography	OAG patients: 135.0 Glaucoma suspects: 135.9	0.92
Jonas 2015 [34]	OAG patients (39) vs. healthy controls (189)	Subfoveal CT	Enhanced depth imaging optical coherence tomography	OAG patients: 241 ± 91 Healthy controls: 258 ± 83	0.18
Sacconi 2017 [35]	OAG patients (35) vs. healthy controls (35)	Subfoveal CT	Spectral-domain optical coherence tomography	OAG patients: 209.9 ± 8.37 Healthy controls: 234.78 ± 8.37	0.042

## Data Availability

No new data were created or analyzed in this study. Data sharing is not applicable to this article.
